# Dual red and near-infrared LED therapy inhibits MRSA biofilm in otitis media

**DOI:** 10.1016/j.bioflm.2025.100314

**Published:** 2025-08-21

**Authors:** Yoo-Seung Ko, Eun-Ji Gi, Sungsu Lee, Hong-Chan Kim, Hyong-Ho Cho

**Affiliations:** Department of Otolaryngology–Head and Neck Surgery, Chonnam National University Medical School and Chonnam National University Hospital, 42 Jaebong-Ro, Dong-Gu, Gwangju, 61469, Republic of Korea

**Keywords:** MRSA, Otitis media, Biofilm, LED therapy, Photobiomodulation, Inflammation

## Abstract

Otitis media (OM), particularly when caused by methicillin-resistant *Staphylococcus aureus* (MRSA), can become refractory due to biofilm formation, which contributes to resistance against conventional antimicrobial treatments. Photobiomodulation using light-emitting diode (LED) therapy has recently emerged as a promising non-antibiotic strategy for managing refractory infections by targeting biofilm-associated pathology. However, especially in the context of MRSA-induced OM, its therapeutic efficacy and underlying mechanisms remain incompletely elucidated. In this study, we established a rat model of OM by inoculating MRSA (5 × 10^8^ CFUs) into the middle ear via the tympanic membrane. Red and near-infrared (NIR) LED irradiation (655/842 nm; 163.2 W/m^2^; 30 min/day for 5 days) was administered 1 week after infection. Scanning electron microscopy revealed a marked reduction in MRSA biofilm structures, and biofilm biomass was significantly decreased, as assessed by crystal violet staining. Quantitative real-time polymerase chain reaction (qRT-PCR) analysis demonstrated significant downregulation of *fib*, *icaB*, *icaC*, and *icaD*, key genes crucial for bacterial adhesion and biofilm development. Histological assessment further showed decreased mucosal thickening and macrophage infiltration, supported by reduced ionized calcium-binding adapter molecule 1 (Iba1) expression. These findings suggest that dual red and NIR LED therapy effectively suppresses MRSA biofilm formation and inflammation in OM, indicating its potential as a novel non-antibiotic therapy for biofilm-associated OM that may help manage persistent or treatment-resistant cases in clinical settings.

## Highlights

• Red/NIR LED irradiation suppresses mucosal thickening and macrophage infiltration in OM.
• Biofilm structure and biomass are significantly reduced by Red/NIR LED therapy in OM.
• Dual-wavelength LED light downregulates MRSA biofilm-related gene expression.
• Red/NIR LED therapy reduces biofilm and inflammation in MRSA-induced OM.
• Red/NIR LED shows potential as a non-antibiotic approach for treating OM biofilm.

## 1 Introduction

Otitis media (OM), an inflammatory condition of the middle ear (ME), is primarily caused by bacterial infections and most commonly presents as acute otitis media (AOM). While the majority of AOM cases resolve spontaneously or respond to appropriate medical therapy, recent studies suggest that biofilm formation can occur even in AOM, contributing to persistent inflammation and treatment failure [1,2]. These biofilms protect bacteria from both host immune responses and antibiotics, reducing treatment efficacy and increasing the risk of progression to recurrent, intractable, or chronic forms of OM. In such cases, patients may experience persistent otorrhea, frequent exacerbations, and poor response to conventional therapies, ultimately leading to prolonged disease and considerable impairment in quality of life. Furthermore, inadequately treated OM can progress to severe intratemporal or intracranial complications, including facial nerve paralysis, labyrinthitis, mastoiditis, meningitis, and epidural, subdural, or brain abscess formation, which may be life-threatening [3,4]. Beyond the clinical burden, recurrent or refractory OM also poses a significant public health concern due to excessive antibiotic use. OM is the most common reason for antibiotic prescriptions in children, with a reported prescribing rate of 85.6 % in the pediatric population [5]. This overreliance on antibiotics has contributed significantly to the emergence of multidrug-resistant pathogens such as methicillin-resistant *Staphylococcus aureus* (MRSA). First identified in 1961, MRSA has become increasingly prevalent, complicating treatment strategies and limiting therapeutic options [6].

Biofilm formation has been identified as a key underlying mechanism in many refractory cases. Recent studies demonstrate that biofilm formation by common otopathogens (e.g., *Haemophilus influenzae*, *Streptococcus pneumoniae*, *S. aureus*, *Moraxella catarrhalis*) is crucial for the pathogenesis of refractory and recurrent OM [7]. These biofilms are structured microbial communities enclosed within an extracellular polymeric substance (EPS) matrix, enhancing their antibiotic resistance. Biofilms confer multidrug resistance and enable bacteria to evade host immune responses while surviving various environmental stresses, further complicating treatment [8,9]. Unlike planktonic bacteria, biofilm-associated microorganisms exhibit distinct phenotypic traits, including altered gene expression and growth rates, contributing to increased resistance to antimicrobial agents and immune defenses. Biofilm formation involves the irreversible attachment of bacterial cells to surfaces or one another within the EPS matrix, making them particularly difficult to eradicate. Consequently, antibiotics are often ineffective in managing refractory or recurrent OM, regularly requiring surgical intervention as a last resort. Therefore, novel therapeutic strategies that target biofilm-associated OM are urgently required.

Given the limitations of conventional antibiotics, alternative therapeutic strategies have been explored. For example, anti-DNABII Fab reduces extracellular DNA (eDNA), a key structural component of biofilms, while the Hydrodebrider System and the photosensitizer Chlorin e6 effectively disrupt biofilms and enhance antibacterial activity, respectively [[10], [11], [12]]. However, these approaches have limitations, including potential cytotoxicity, the need for repeated applications, and incomplete biofilm eradication, highlighting the ongoing challenges in biofilm-targeted therapies [13].

Photobiomodulation (PBM), a noninvasive therapeutic approach, utilizes low-level light, typically emitted by lasers or light-emitting diodes (LEDs), to regulate inflammation, promote tissue repair, and modulate microbial growth [14]. Compared to laser-based PBM, LED-based PBM offers a safer, more accessible modality with similar therapeutic outcomes. Recently, PBM has attracted attention for its potential in managing biofilm-associated infections. While studies report the benefits of PBM in immune modulation and wound healing, the precise mechanisms underlying its effects on bacterial biofilms remain unclear [[15], [16], [17]]. PBM exerts antimicrobial effects through multiple pathways, including the production of reactive oxygen species (ROS), disruption of the biofilm matrix, and alteration of the expression of bacterial gene related to adhesion and persistence. Studies indicate that red (600–700 nm) and near-infrared (NIR) (770–1200 nm) wavelengths reduce inflammation, accelerate wound healing, and inhibit bacterial growth. In our previous study, we reported that dual red and NIR LED irradiation effectively alleviated inflammation in a rat model of lipopolysaccharide-induced OM, suggesting its potential as a noninvasive therapeutic strategy [18].

Despite growing interest in PBM, its effects on MRSA-induced OM remain largely underexplored, particularly with respect to biofilm formation. Given the critical role of biofilms in treatment-resistant infections, elucidating the molecular mechanisms through which PBM influences biofilm development could provide valuable insights for optimizing this therapeutic approach. Based on existing evidence, we hypothesize that dual red and NIR LED irradiation inhibits MRSA-induced biofilm formation by disrupting the biofilm matrix and modulating bacterial gene expression. To test this hypothesis, we evaluate the inhibitory effects of red and NIR LED irradiation on MRSA-induced biofilm formation in a rat model of OM. Therefore, this study aims to investigate PBM's underlying mechanisms and assess its potential as a non-antibiotic therapeutic strategy to prevent biofilm persistence and infection recurrence in OM by analyzing biofilm structural integrity and the expression of key biofilm-related genes.

## 2 Materials and methods

### 2.1 Sex as a biological variable

Male Sprague-Dawley rats were exclusively used in this study to minimize hormonal fluctuations and reduce inter-individual variability, thereby enabling more consistent OM modeling. Although the use of male animals enhances reproducibility, it may limit the generalizability of the findings, given the possibility of sex-based differences in immune and inflammatory responses. Thus, future studies should assess potential sex-specific responses to LED therapy.

### 2.2 Animal model and experimental induction of OM

Sprague-Dawley rats (age: 4–5 weeks; weight: 200–250 g) were obtained from Damul Science (Daejeon, Republic of Korea). The rats were housed with adequate food and water, and all experiments were conducted in strict compliance with Chonnam National University's Guide for the Care and Use of Laboratory Animals. The study protocol was approved by the university's Committee on the Ethics of Animal Experiments (CNUHIACUC-21047). All rats remained alive throughout the study, and normal tympanic membranes were confirmed before MRSA injection. Anesthesia was induced with intraperitoneal injections of ketamine (100 mg/kg) and xylazine (10 mg/kg) prior to MRSA injection and LED irradiation as well as before euthanization. The OM animal model was established by injecting MRSA (5 × 10^8^ colony-forming units (CFUs)) into the ME of the rats through the tympanic membrane, with controls injected with phosphate-buffered saline (PBS). Seven days after MRSA injection, the rats with OM were randomly assigned to two groups: the red and NIR LED-irradiated group and the non-irradiated group.

### 2.3 Bacterial strain and culture conditions

The MRSA strain ATCC 33591 used in this study was cultured on tryptic soy agar (Kisan Bio, Seoul, Republic of Korea) at 37 °C for 24 h and stored at 4 °C for future use. For *in vitro* assays, MRSA was cultured in tryptic soy broth and incubated in an orbital shaker (160 rpm) at 37 °C overnight. The strain was subcultured every 3–4 weeks to maintain its viability. For long-term storage, bacterial stocks were preserved in glycerol at −80 °C.

### 2.4 Antibodies

A polyclonal rabbit anti- Iba1 antibody was obtained from FUJIFILM Wako Pure Chemical Corporation (Osaka, Japan). For immunofluorescence, a donkey anti-rabbit IgG Alexa Fluor 488 (H + L) antibody was purchased from Jackson ImmunoResearch (West Grove, PA, USA) and used as the secondary antibody.

### 2.5 Light source and irradiation protocol

A continuous-wave dual red and NIR LED irradiation system (HK Healthcare Co., Ltd., Gwangju, Republic of Korea) with wavelengths of 655 nm and 842 nm was used. These wavelengths were selected based on their established efficacy in previous PBM studies targeting bacterial inflammation [18]. The system's configuration—including the control module, battery, and light source—was previously described and illustrated in the supplementary material of our earlier study [18]. The LED light source unit included both an LED that emitted light with a power intensity of 163.2 W/m^2^ and an optical fiber. To assess the therapeutic effects of red and NIR LED irradiation on MRSA-induced OM, rats were exposed to irradiation through the ear canal for 30 min per day over 5 consecutive days, starting on day 7 after MRSA injection. The optical fiber (3 mm in diameter) was securely placed in the cartilaginous portion of the external auditory canal, with its orientation carefully adjusted to ensure proper alignment with the tympanic membrane during irradiation. In the control group, the optical fiber was placed in the same position under identical anesthesia conditions, but the LED light source remained inactivated. The irradiation distance from the optical fiber to the tympanic membrane was maintained at 0.5 cm, resulting in an effective power density of 163.2 W/m^2^. The total energy delivered over 30 min was 293,760 J/m^2^, which corresponds to a fluence of 29.376 J/cm^2^. Supplementary Table 1 summarizes the detailed irradiation parameters, including wavelengths, radiant intensity, exposure time, energy, and fluence.

### 2.6 Histopathological analyses

Following euthanasia, the tympanic bullae were harvested and fixed in 4 % paraformaldehyde at 4 °C for 24 h. Subsequently, the samples were rinsed with PBS and decalcified in Calci-Clear Rapid (National Diagnostics, Atlanta, GA, USA) for 5 days. Once softened, the bullae were dehydrated and embedded in paraffin, after which they were sectioned into 7-μm longitudinal slices for staining. The sections were deparaffinized, rehydrated, and stained with hematoxylin and eosin (H&E) to visualize the ME mucosa.

### 2.7 Immunofluorescence staining

The sections were washed three times with PBS supplemented with 0.1 % Triton X-100, blocked with 5 % donkey serum for 1 h at room temperature, and incubated overnight at 4 °C with rabbit anti-Iba1 antibody (1:500 dilutions, Wako #019–19741) as the primary antibody. They were then incubated at room temperature for 1 h with an Alexa Fluor 488-conjugated secondary antibody, donkey anti-rabbit (1:500, Jackson ImmunoResearch, catalog #711-545-152). To label cell nuclei, all sections were counterstained with 4′,6-diamidino-2-phenylindole (1: 10,000; Invitrogen, Carlsbad, CA, USA, catalog #MP01306) for 10 min at room temperature. Staining and visualization were performed using a Zeiss LSM 510-META confocal laser scanning microscope (Zeiss, Oberkochen, Germany). Iba1-positive cells in the ME were quantified using ZEN Microscopy Software (Zeiss). The results are presented as bar graphs with values as means ± SEM (n = 8).

### 2.8 Quantitative analysis of biofilm-related gene expression using qRT-PCR

Total RNA was extracted from the ME mucosa of rats using TRIzol reagent (Invitrogen) following the manufacturer's instructions. RNA samples were treated with DNase I (Merck, Darmstadt, Germany) to remove genomic DNA contamination. Complementary DNA was synthesized from 1 μg of RNA using the PrimeScript™ RT Reagent Kit (Takara, Kyoto, Japan). Quantitative real-time polymerase chain reaction (qRT-PCR) was performed using SYBR® Green PCR Master Mix (Takara) and monitored with the Thermal Cycler Dice® Real-Time System III (Takara). Primers were designed to amplify target endogenous genes, with 16S rRNA serving as the internal control. The stability of 16S rRNA expression was confirmed across all treatment groups prior to its use as a reference gene. Target genes' relative expression levels were calculated using the ΔΔCt method: 2^−(ΔCt_target gene − ΔCt_16S rRNA). All reactions were performed in triplicate. Supplementary Table 2 provides the primer sequences.

### 2.9 SEM

Mice were euthanized, and their hearts were perfused with 4 % paraformaldehyde. The fixed tympanic bullae were isolated and immersed in 2.5 % glutaraldehyde in PBS overnight at 4 °C. Subsequently, the samples were rinsed with PBS and decalcified in 5 % EDTA at 4 °C for 5 days. Post-fixation was performed in 1 % OsO_4_ for 2 h at room temperature. The samples were dehydrated in ethanol at different concentrations, critical point dried, sputter-coated with metal, and examined using an EM-30AX Plus COXEM scanning electron microscope (SEM, NI Tech Co., Ltd., Yongin, Republic of Korea). SEM imaging was conducted at an accelerating voltage of 15 kV with a working distance of 12.6 mm, and representative images were acquired at a magnification of × 500.

### 2.10 Biofilm disruption and bacterial recovery methods

After the harvested bullae were gently rinsed with sterile PBS to remove non-adherent cells and debris, the tissues were immersed in 15 mL of sterile PBS (pH 7.4). The samples were then subjected to ultrasonic treatment at a frequency of 35–40 kHz for 5–10 min. During sonication, the samples were shaken every 30 s to ensure uniform processing while maintaining a temperature <37 °C. After sonication, the sample solution was transferred to a sterile tube and centrifuged at 5000 rpm for 5 min to pellet the bacteria. The supernatant was discarded, and the pellet was resuspended in sterile PBS for further analysis. To facilitate additional biofilm removal, DNase I (Merck) was added to the sample at a final concentration of 50–100 μg/mL. The enzyme activity was enhanced by supplementing the reaction with 10 mM MgCl_2_ or 5 mM CaCl_2_, as required. The mixture was gently shaken and incubated at 37 °C for 15–30 min. The recovered bacteria were diluted to concentrations ranging from 1 × 10^5^ to 5 × 10^6^ and plated on tryptic soy agar, and CFU counts were determined. Biofilm disruption was performed following previously established protocols [19,20] to ensure consistency and reliability. Although a standard biofilm-disrupting control agent was not used, CFU reductions post-treatment provided indirect validation of biofilm removal efficacy.

### 2.11 Crystal violet staining for biofilm quantification

After the collected bullae were gently rinsed with sterile PBS to remove non-adherent cells and debris, the tissues were stained with a 1 % crystal violet solution for 20 min at room temperature. Following this, the excess dye was removed by washing the tissues with PBS until no unbound stain remained, and the tissues were allowed to air dry completely. To quantify the biofilm, the bound crystal violet dye was solubilized by adding 95 % ethanol to each sample, followed by incubation with shaking for 10–30 min. The absorbance of the solubilized dye was measured at 570 nm using a spectrophotometer. To ensure accurate quantification, background absorbance from non-specific staining (e.g., tissue alone without biofilm) was subtracted from each measurement. All experiments were performed in triplicate, with absorbance values averaged. Statistical analyses were conducted to compare biofilm formation between experimental groups.

### 2.12 Statistical analyses

Statistical analyses were performed using SPSS version 27 (Chicago, IL, USA). Nonparametric tests, including the Mann–Whitney U and Kruskal–Wallis tests, were used to assess statistical significance. For datasets that met the assumption of normality (based on sample sizes of n = 6 or 8), one-way ANOVA followed by Tukey's HSD post hoc test or Student's t-test was applied. Normality was assessed using the Kolmogorov–Smirnov and Shapiro–Wilk tests. A p-value of less than 0.05 was considered statistically significant.

## 3 Results

### 3.1 Induction of OM in rats using MRSA

To verify the successful induction of OM using MRSA in the rat model, an otoscopic examination of the tympanic membrane was performed. Fig. 1A illustrates that the tympanic membrane in the control group remained transparent, exhibiting no signs of inflammation or infection. In contrast, the MRSA-infected group (Fig. 1B–E) exhibited a progressive inflammatory response starting 1 day after MRSA inoculation. The inflammation gradually resolved by day 11; however, histopathological analysis revealed a sustained increase in mucosal thickness in the ME (Fig. 1F–I). This sustained thickening of the ME mucosa suggests tissue hypertrophy, potentially due to prolonged inflammatory stimulation caused by MRSA infection. Such persistent mucosal hypertrophy may reflect subclinical inflammation that increases the ME's susceptibility to recurrent episodes or prolonged disease. Additionally, histopathological examination (Fig. 1F–I) revealed structural alterations in the ME, including epithelial hyperplasia and inflammatory cell infiltration, suggesting that MRSA infection may induce persistent tissue changes that could contribute to prolonged or recurrent ME pathology.

**Fig. 1:**
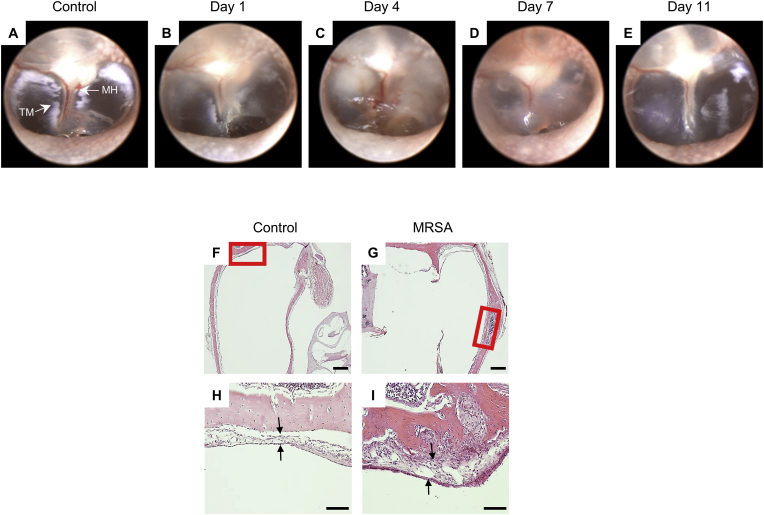
**Establishment of rat model of OM induced by MRSA injection through tympanic membrane.** (A–E) Otoscopic images of tympanic membrane following MRSA injection. (F–G) H&E staining of ME at 2.5 × magnification. (H–I) H&E staining at 20 × magnification. Scale bars: 500 μm (F–G); 100 μm (H–I). Abbreviations: MH, malleus handle; TM, tympanic membrane.

### 3.2 Reduction of ME mucosal thickness using LED irradiation

**Fig. 2:**
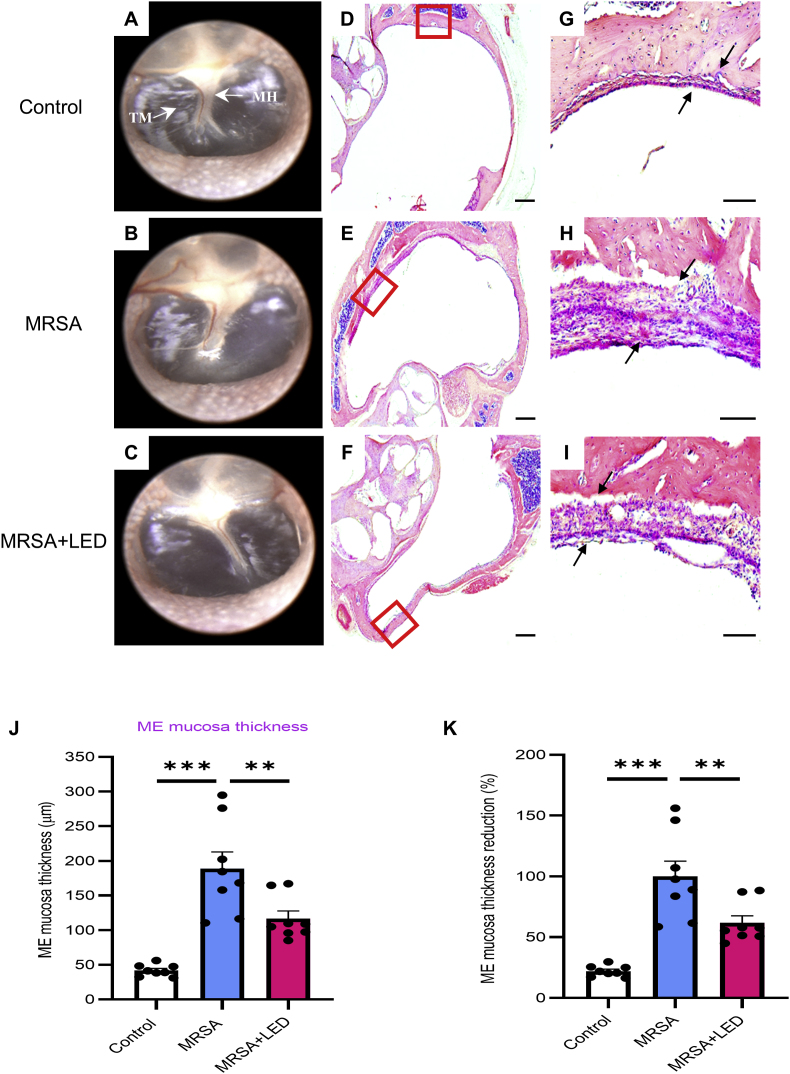
**Red and NIR LED irradiation reduces ME mucosal thickening induced by MRSA infection**. (A–C) Representative otoscopic images of tympanic membrane from MRSA-infected rats with or without LED irradiation on day 11. (D–F) H&E staining of ME tissue showing histopathological changes in MRSA-infected rats with or without LED irradiation at 2.5 × magnification. (G–I) H&E staining at 20 × magnification. Scale bars: 500 μm (D–F); 100 μm (G–I). (J) Quantification of ME mucosal thickness in selected regions (n = 8). (K) Percentage reduction in ME mucosal thickness relative to MRSA-induced OM group. Data are expressed as means ± SEM. One-way ANOVA: ∗p < 0.05, ∗∗p < 0.01, ∗∗∗p < 0.001. Abbreviations: MH, malleus handle; TM, tympanic membrane. (For interpretation of the references to colour in this figure legend, the reader is referred to the Web version of this article.)

Rats were exposed to red and NIR irradiation through the ear canal to investigate its therapeutic effects on MRSA-induced OM. The irradiation was delivered at wavelengths of 655 nm and 842 nm with an intensity of 163.2W/m^2^ for 5 consecutive days, starting on day 7 post-MRSA injection. Fig. 2A–C illustrates that the otoscopic examination on day 11 revealed the resolution of the inflammation, with no significant differences observed between the groups. However, to further evaluate the therapeutic effects of LED irradiation, we examined its influence on the thickness of the ME mucosa, which had increased due to MRSA-induced inflammation. Increased ME mucosal thickness is often associated with persistent inflammation, biofilm formation, and chronic infections. ME mucosal thickness was considerably greater in the MRSA-induced OM group than in the control group, accompanied by a significant increase in inflammatory cell infiltration. In contrast, LED irradiation significantly reduced both ME mucosal thickness and inflammatory cell infiltration. A comparison between the irradiated and non-irradiated MRSA-induced OM groups showed that the reduction in mucosal thickness following LED treatment was statistically significant, suggesting a potential anti-inflammatory effect (Fig. 2D–I). Specifically, the ME mucosal thickness in the control group measured 41.7 ± 3.0 μm, which increased to 188.8 ± 23.8 μm in the MRSA-induced OM group. However, in the red and NIR LED-irradiated OM group, the ME mucosal thickness was significantly reduced to 116.5 ± 11.1 μm, corresponding to a 38.3 % reduction compared to the MRSA-induced OM group (Fig. 2J and K).

**Fig. 3:**
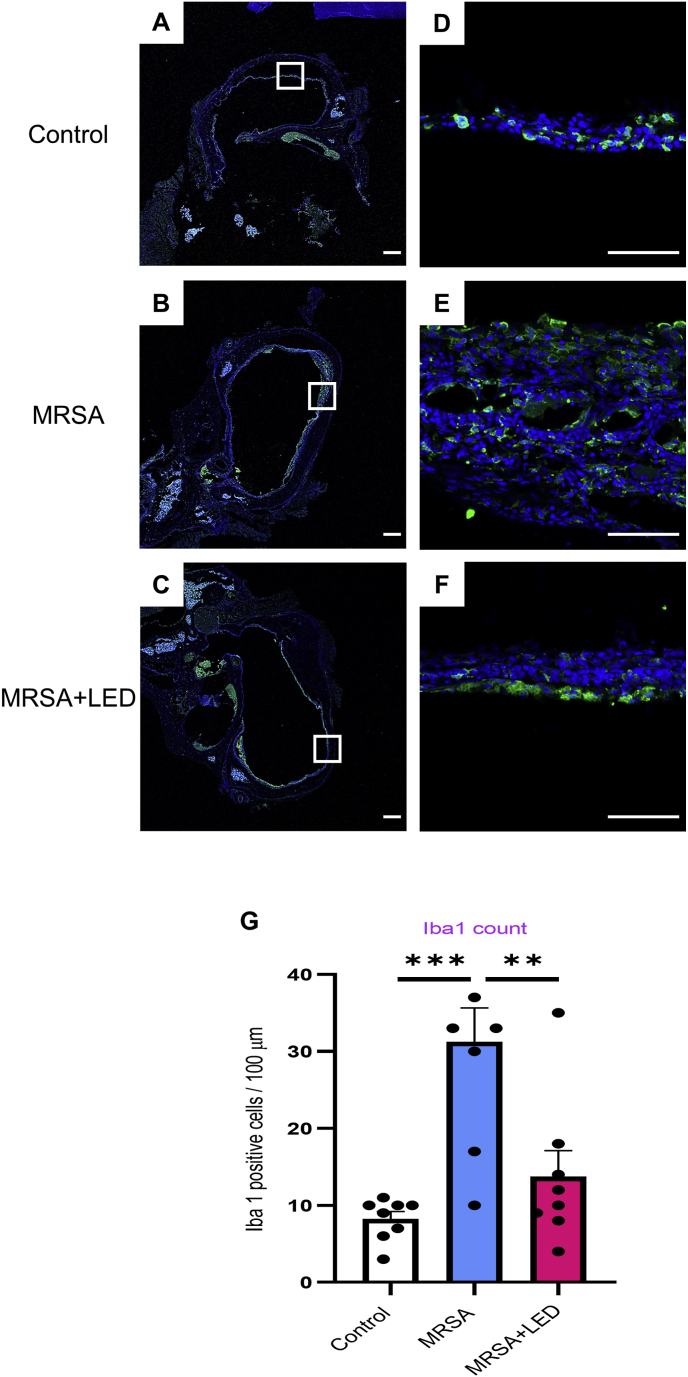
**Red and NIR LED irradiation reduces MRSA-induced macrophage activation and expression of Iba1 in ME.** (A–C) Representative immunofluorescence images of ME tissue from MRSA-infected rats with or without LED irradiation on day 11, shown at 2.5 × magnification. (D–F) Higher magnification (20 × ) immunofluorescence images highlighting Iba1 expression. Scale bars: 500 μm (A–C); 100 μm (D–F). (G) Quantification of Iba1-positive cells per 100 μm in selected regions (n = 8). Data are expressed as means ± SEM. One-way ANOVA: ∗p < 0.05, ∗∗p < 0.01, ∗∗∗p < 0.001. (For interpretation of the references to colour in this figure legend, the reader is referred to the Web version of this article.)

Since ME mucosal thickening is often associated with inflammatory cell infiltration, the specific cell types present in the thickened ME tissue were examined. To further investigate the anti-inflammatory effects of LED irradiation at the cellular level, inflammatory cell activation was assessed using immunofluorescence staining for Iba1, an activated macrophage marker, where Iba1-positive cells served as inflammation and macrophage activation markers. LED irradiation significantly reduced the number of Iba1-positive cells in the ME (Fig. 3A–F). In the control group, the number of Iba1-positive cells was 8.2 ± 0.8, increasing to 31.2 ± 4.1 in the MRSA-induced OM group. However, in the red and NIR LED-irradiated OM group, the number of Iba1-positive cells was significantly reduced to 13.7 ± 3.1 (Fig. 3G), suggesting that LED irradiation effectively suppresses inflammatory cell activation in the ME. These findings suggest that MRSA-induced OM in rats resulted in a marked increase in ME mucosal thickness and inflammatory cell infiltration, both of which were noticeably reduced by red and NIR LED irradiation. This effect may be attributed to the modulation of inflammatory cell activity and a reduction in macrophage presence in the ME.

### 3.3 Inhibition of biofilm formation using LED irradiation

**Fig. 4:**
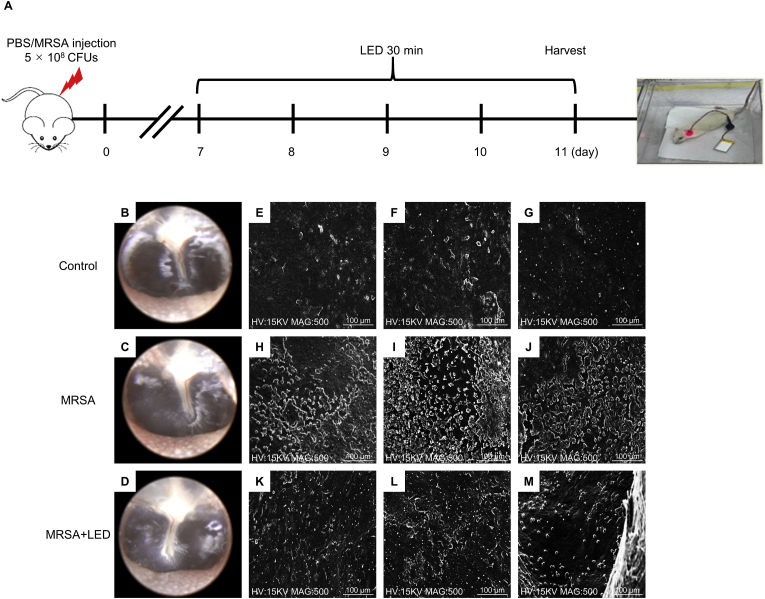
**Red and NIR LED suppresses biofilm formation in MRSA-induced ME.** (A) Schematic diagram illustrating experimental timeline, including MRSA injection and LED treatment schedule. (B–D) Otoscopic images of tympanic membrane on day 11 showing no visible differences between MRSA-infected rats with or without LED irradiation. (E–G) SEM images of ME from control group. (H–J) SEM images of non-irradiated MRSA-induced group. (K–M) SEM images of LED-irradiated MRSA-induced group. Each panel (E–G; H–J; K–M) shows representative samples from the same group, illustrating variability in biofilm formation. Scale bars: 100 μm (E–M). (For interpretation of the references to colour in this figure legend, the reader is referred to the Web version of this article.)

**Fig. 5:**
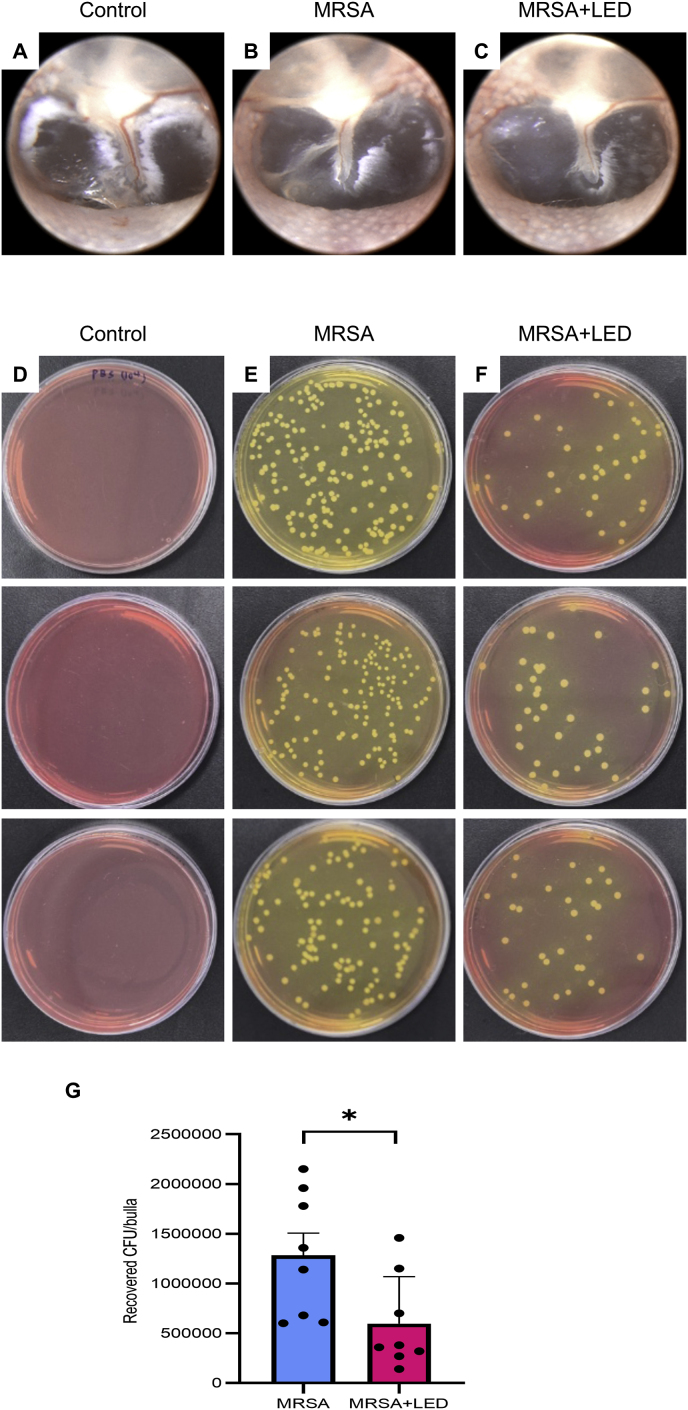
**Red and NIR LED irradiation reduces MRSA colonization in ME.** (A–C) Otoscopic images of tympanic membrane on day 11. (D–F) Representative agar plates showing MRSA colonies cultured from bullae, including cochleae, in each experimental group. (G) Quantification of MRSA CFUs showing significant reduction in bacterial colonization in LED-irradiated group compared to non-irradiated MRSA group (n = 8). Data are expressed as means ± SEM. Statistical analysis: one-way ANOVA; ∗p < 0.05, ∗∗p < 0.01, ∗∗∗p < 0.001. (For interpretation of the references to colour in this figure legend, the reader is referred to the Web version of this article.)

SEM is the preferred method for visualizing biofilms when high magnification and resolution are necessary for accurate morphological characterization [[21], [22], [23]]. This tool is particularly useful in comparative studies evaluating the anti-biofilm efficacy of drugs or treatments, as SEM results consistently align with findings from other analytical methods [[24], [25], [26]]. In this study, the effects of red and NIR LED irradiation on biofilm formation were evaluated in a rat model of MRSA-induced OM (Fig. 4A). Representative otoscopic images taken on day 11 revealed no significant differences in tympanic membrane appearance between MRSA-induced rats with and without LED irradiation, as inflammation had already resolved by this time (Fig. 4B–D). However, SEM analysis of ME tissues revealed significant differences in biofilm formation among the groups. In the non-irradiated MRSA-induced group, a dense and well-structured biofilm was observed, indicating robust biofilm formation that was considerably more extensive than that in the control group. In contrast, the LED-irradiated group exhibited a notable reduction in biofilm density, with a more scattered and disrupted structure (Fig. 4E–M). Consistent with these findings, the number of MRSA CFUs recovered from ME tissues was substantially lower in the LED-irradiated group than in the non-irradiated MRSA-induced group (Fig. 5A–G). These findings imply that LED irradiation inhibits biofilm development and effectively reduces bacterial survival. Despite the resolution of inflammatory signs in the tympanic membrane following MRSA-induced OM, biofilm formation persists in the ME, posing an ongoing risk of chronic infection. Nevertheless, these findings demonstrate that red and NIR LED irradiation substantially reduces biofilm formation and bacterial survival, highlighting its potential as a therapeutic strategy for biofilm-associated conditions, such as OM.

### 3.4 Reduction of biofilm formation and mass using LED irradiation

**Fig. 6:**
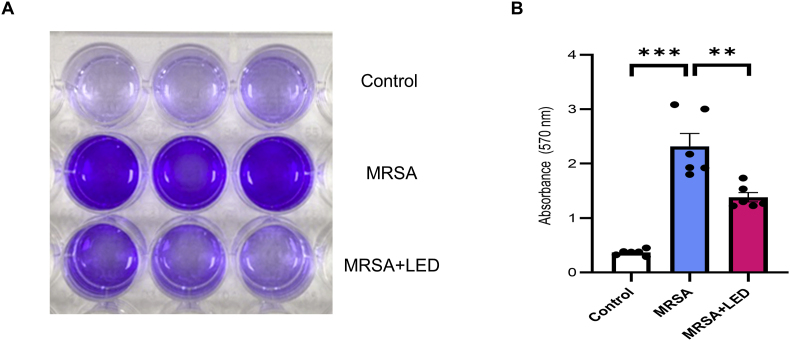
**Red and NIR LED irradiation reduces biofilm biomass in MRSA-induced OM.** (A) Representative images of crystal violet-stained biofilms in ME of non-irradiated and LED-irradiated groups showing visibly reduced biofilm biomass in LED-treated group. (B) Quantitative analysis of crystal violet staining intensity, indicating significant reduction in biofilm biomass in LED-treated group (n = 6). Data are expressed as means ± SEM. Statistical analysis: one-way ANOVA; ∗p < 0.05, ∗∗p < 0.01, ∗∗∗p < 0.001. (For interpretation of the references to colour in this figure legend, the reader is referred to the Web version of this article.)

To evaluate the biofilm mass in MRSA-induced OM, crystal violet staining was performed on the bullae harvested from both the LED-irradiated and non-irradiated groups. On day 11, post-MRSA injection, the bullae were collected for biofilm assessment. Crystal violet staining confirmed a significant reduction in biofilm mass following red and NIR LED irradiation (Fig. 6A). Absorbance measurements at 570 nm were significantly lower in the LED-irradiated group than in the non-irradiated group, indicating a decrease in biofilm biomass (Fig. 6B). These results are consistent with those of SEM imaging, demonstrating a reduction in biofilm density. Collectively, these findings suggest that red and NIR LED irradiation effectively reduces biofilm formation in MRSA-induced OM, supporting its potential as a noninvasive therapeutic strategy for biofilm-associated infections.

### 3.5 Downregulation of key biofilm-associated genes using LED irradiation

**Fig. 7:**
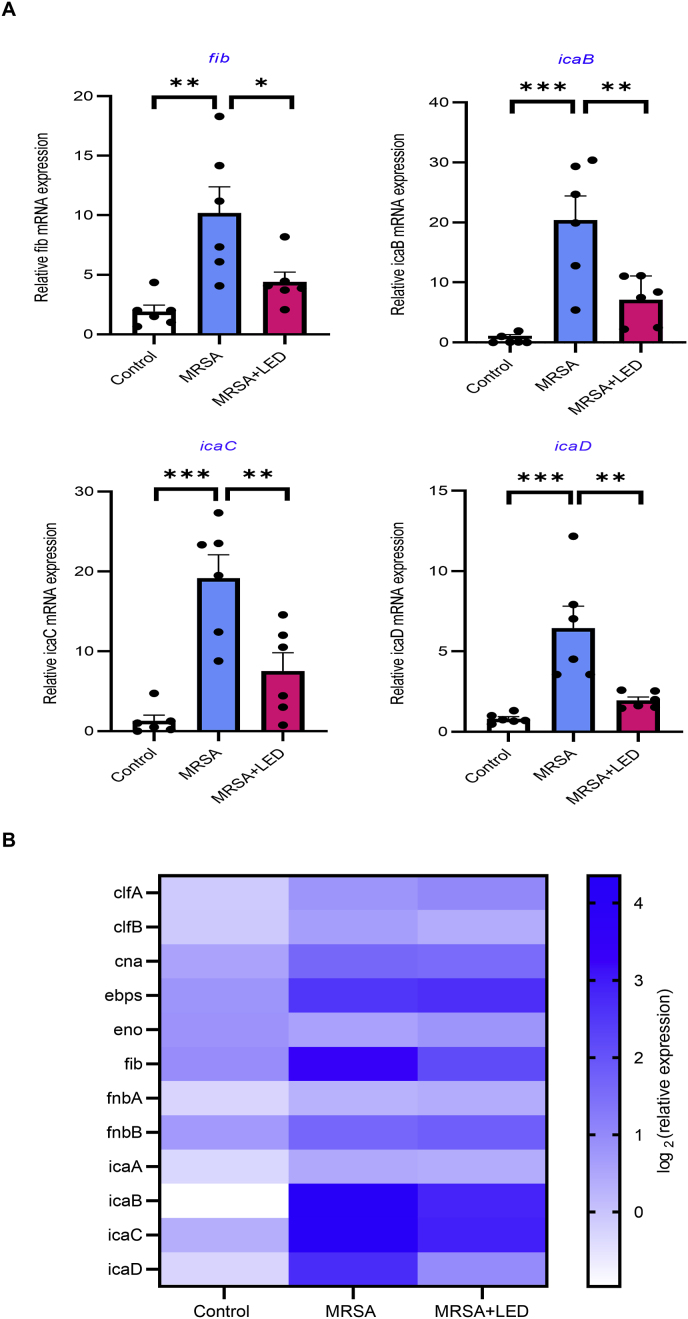
**Red and NIR LED irradiation downregulates key biofilm-associated gene expression in MRSA-infected ME.** (A) qRT-PCR analysis of gene expression in ME of MRSA-infected rats. LED-irradiated groups showed significantly reduced RNA levels of *fib*, which mediates bacterial adherence, and *icaB, icaC,* and *icaD*, which are involved in biofilm formation (n = 6). Data are expressed as means ± SEM. Statistical analysis: one-way ANOVA; ∗p < 0.05, ∗∗p < 0.01, ∗∗∗p < 0.001. (For interpretation of the references to colour in this figure legend, the reader is referred to the Web version of this article.)

**Fig. 8:**
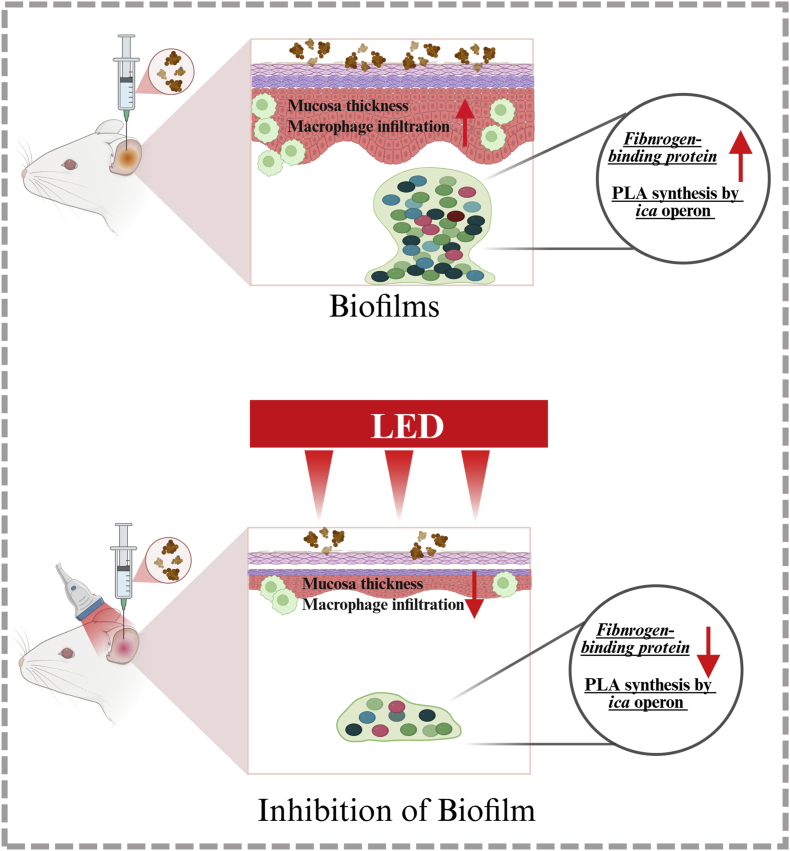
**Therapeutic effects of red and NIR LED in rat model of MRSA-induced OM.** Schematic diagram summarizing potential mechanisms underlying red and NIR LED therapy in reducing biofilm formation, decreasing inflammation, and improving pathological outcomes in MRSA-induced OM. (For interpretation of the references to colour in this figure legend, the reader is referred to the Web version of this article.)

Biofilm formation in *S. aureus* is regulated by multiple genes involved in adhesion, aggregation, and extracellular matrix production. Among these, *fib* encodes fibrinogen-binding protein (Fib), a key factor in bacterial adhesion and biofilm stabilization. Fib mediates the binding of *S. aureus* to fibrinogen, facilitating cell–cell aggregation and enhancing biofilm formation. Additionally, *fib* facilitates immune evasion by interacting with host fibrinogen, enabling *S. aureus* to establish persistent infections. The *ica* operon, composed of *icaA*, *icaB*, *icaC*, and *icaD*, regulates the synthesis of polysaccharide intercellular adhesin (PIA), a key structural component of the biofilm matrix that promotes bacterial aggregation and enhances protection against environmental stressors. To further investigate the molecular mechanisms responsible for the biofilm mass reduction following red and NIR LED irradiation, qRT-PCR analysis was performed to analyze the expression of biofilm-associated genes. The results revealed a significant downregulation of *fib*, *icaB*, *icaC*, and *icaD* in the LED-irradiated group compared to the non-irradiated group (Fig. 7A). The decreased expression of *fib* suggests that LED treatment disrupts fibrinogen-mediated bacterial aggregation, thereby inhibiting biofilm formation (Fig. 7A). Additionally, the downregulation of *icaB*, *icaC*, and *icaD* indicates a decrease in the synthesis of PIA, a key factor in biofilm stability and adhesion. Fig. 7B presents a heatmap summarizing the relative expression patterns of these genes across experimental groups and illustrating specific gene responses to LED treatment. These findings are consistent with the biofilm mass reduction observed in crystal violet staining and SEM imaging, further supporting the inhibitory effect of LED irradiation on biofilm formation at the genetic level. In contrast, the expression levels of other biofilm- and adhesion-related genes (*clfA, clfB, cna, ebpS, eno, fnaA, fnaB, and icaA;* Supplementary Fig. 1) remained unchanged after LED treatment. Supplementary Table 2 provides the primer sequences used for qRT-PCR analysis. Fig. 8 presents a schematic illustration of the therapeutic effects of red and NIR LED irradiation on MRSA-induced biofilm formation in our rat model of OM. The diagram highlights how LED irradiation selectively downregulates key biofilm-related genes, particularly those involved in bacterial adhesion and biofilm matrix production. This genetic modulation leads to reduced biofilm formation, decreased inflammation, and potentially improved outcomes in biofilm-associated or treatment-resistant OM. Our findings suggest that red and NIR LED irradiation is a noninvasive and effective strategy for controlling biofilm-associated infections by targeting key molecular pathways involved in bacterial adhesion and aggregation.

## 4 Discussion

This study demonstrated that dual red and NIR LED irradiation effectively reduced MRSA-induced inflammation and biofilm formation in a rat model of OM. As biofilm formation is increasingly recognized as a key pathogenic mechanism even in AOM, MRSA-induced OM remains difficult to treat due to its resistance to both host immune defenses and antibiotic therapy [[27], [28], [29]]. While many AOM cases resolve with conventional treatment, the presence of biofilms can lead to recurrent or unresolved infections by shielding bacteria from immune responses and antibiotics [30], ultimately complicating treatment and prolonging the disease course. These challenges underscore the need for safe and effective strategies that specifically target biofilm-associated pathology.

Recent studies have shown that LED irradiation improves tissue perfusion, reduces post-inflammatory hyperpigmentation, accelerates wound healing, and alleviates inflammatory conditions such as arthritis [15,31]. Furthermore, LED therapy inhibits inflammatory cell infiltration and suppresses cytokine expression in various inflammatory models, including collagen-induced tendinitis [32,33]. These findings suggest that LED irradiation may exert broad therapeutic effects by modulating the inflammatory response and promoting tissue regeneration. Beyond its anti-inflammatory effects, LED irradiation exhibits antibacterial activity by generating oxidative stress, disrupting bacterial cell membranes, and inhibiting biofilm formation [34,35]. NIR light has been reported to inhibit biofilms via nitric oxide-mediated photodynamic therapy, which disrupts bacterial membranes, and low-temperature photothermal therapy, which accelerates bacterial death under mild heat [36]. Similarly, red light enhances ROS generation via interfacial charge separation in ZnO-SQ nanohybrids, disrupting biofilm integrity and promoting bacterial eradication [37]. Blue and red light irradiation, for example, reduces the viability of *S. aureus* and *Pseudomonas aeruginosa*, two major pathogens involved in persistent infections [35,38]. These antimicrobial effects suggest that LED therapy may serve as a dual-function treatment by modulating inflammation, promoting tissue regeneration, and reducing bacterial burden in infected tissues. In otologic applications, a 632-nm diode laser has been shown to effectively eradicate *S. pneumoniae*, *H. influenzae*, and *M. catarrhalis* in OM, while an 808-nm diode laser has been used to prevent noise-induced hearing loss [39,40]. Compared to lasers, LED devices provide greater safety, portability, and ease of use, with minimal risk of tissue damage. As a noninvasive approach, LEDs enhance patient comfort and are already widely used in dermatology, pain management, and tissue regeneration. The LED device used in this study was previously shown to suppress inflammation in LPS-induced OM by inhibiting MAPK signaling and pro-inflammatory cytokine production [18]. Here, we evaluated its anti-biofilm effects in MRSA-induced OM.

Our findings demonstrated that MRSA infection induced a robust inflammatory response (Fig. 1), and although overt signs of inflammation resolved by day 11, mucosal thickening persisted (Fig. 2). These sustained structural changes suggest prolonged tissue remodeling and residual inflammation. Similar findings have been reported in previous studies of long-lasting ME inflammation [2,41,42]. Given that mucosal thickening in the ME is often accompanied by inflammatory cell infiltration, we investigated the specific cell types present within the thickened ME tissue.

To investigate the underlying cellular mechanisms, inflammatory cell activation was evaluated using immunofluorescence staining for Iba1, a marker of activated macrophages. This study demonstrated that red and NIR LED irradiation significantly reduced mucosal thickness in the ME (Fig. 2) and macrophage infiltration (Fig. 3). Given that mucosal thickening is closely associated with persistent OM symptoms such as conductive hearing loss and ear fullness, the observed reduction in ME mucosal thickness following LED irradiation suggests a potential therapeutic effect, which may contribute to symptom alleviation. Although we did not conduct behavioral assessments, future investigations incorporating functional evaluations are warranted to further elucidate the clinical relevance of these findings. To characterize the inflammatory profile in more detail, myeloperoxidase (MPO) staining was conducted to detect neutrophils, which are primarily involved in the early phase of inflammation. However, no MPO-positive cells were detected, suggesting that neutrophil activity had already subsided by day 11 post-MRSA injection, when the tissue was collected. This time point corresponds to a later stage of the acute inflammatory response, during which macrophages typically become the predominant immune cell type. Consistent with this finding, immunofluorescence staining for Iba1 revealed prominent macrophage infiltration in the infected ME. This cellular profile, characterized by reduced neutrophils and increased macrophages, likely reflects a sustained acute inflammatory response. The extended inflammatory activity observed in this model may be attributed to the virulence of the MRSA strain used, which can intensify and prolong immune activation. Biofilm formation plays a key role in the persistence of OM and the resistance of MRSA to conventional antibiotic therapies [[27], [28], [29]].

In this study, SEM analysis revealed dense biofilm structures within the ME of MRSA-infected rats (Fig. 4), consistent with previous studies demonstrating the long-term presence of biofilms in OM despite the resolution of visible inflammation [43,44]. However, in the LED-irradiated group, biofilm density was substantially reduced, with the biofilm appearing more dispersed (Fig. 4), leading to a decrease in recoverable MRSA colonization in the ME (Fig. 5). This finding aligns with those of previous studies showing that PBM disrupts biofilm architecture and reduces its overall density [[45], [46], [47]]. Additionally, one study indicated that NIR irradiation, when combined with l-arginine, indocyanine green, and mesoporous polydopamine, generates heat and ROS, thereby disrupting biofilms through synergistic photodynamic and photothermal mechanisms [36].

Moreover, quantitative crystal violet staining was found to significantly reduce biofilm biomass in the LED-treated group, further validating the anti-biofilm efficacy of LED therapy (Fig. 6). Studies show that the photo-induced release of nitric oxide and its derivatives within the EPS matrix disrupts biofilms by inducing nitrosative stress and interfering with the quorum sensing pathways, ultimately promoting biofilm dispersal [37,[48], [49], [50]]. Our findings suggest that LED therapy disrupts biofilm architecture and potentially interferes with bacterial communication pathways that contribute to biofilm stability. These results highlight the therapeutic potential of LED irradiation in mitigating biofilm formation, addressing a major challenge in the treatment of bacterial infections such as MRSA-induced OM.

The *icaADBC* operon encodes enzymes responsible for synthesizing PIA, a key component of the biofilm matrix that facilitates bacterial adhesion and contributes to antibiotic resistance [51,52]. Similarly, the *fib* gene is crucial for bacterial adhesion to host tissues, supporting biofilm establishment and persistence [53]. In this study, LED irradiation significantly downregulated the expression of key biofilm-associated genes, including *fib*, *icaB*, *icaC*, and *icaD*, in the LED-treated group (Fig. 7). This finding suggests that LED therapy reduces biofilm biomass and disrupts the genetic pathways essential for biofilm development. The observed reduction in gene expression indicates that LED irradiation disrupts both the structural integrity of biofilms and the molecular pathways essential for their formation. However, the downregulation of these genes alone may not fully explain the extent of biofilm reduction, implying that additional mechanisms—beyond gene expression changes—may contribute to LED therapy's anti-biofilm effects**.**

This study provides strong evidence that red and NIR LED irradiation effectively inhibits biofilm formation, demonstrating its therapeutic potential for managing MRSA-induced OM. Compared to conventional treatments, LED therapy offers several advantages, including noninvasiveness, deep tissue penetration, and a favorable safety profile with minimal reported adverse effects. It may enhance the efficacy of conventional treatments, such as antibiotics, by reducing biofilm formation, thereby addressing a key obstacle in the treatment of antibiotic-resistant OM. Despite these promising results, this study has several limitations. First, while the rat model offers valuable insights into OM pathophysiology, it may not fully replicate the complexity of human ME infections. Moreover, though advantageous for experimental reproducibility and cross-study comparability, the use of a standardized MRSA strain (ATCC 33591) may not adequately reflect the genetic and phenotypic diversity observed in clinical MRSA isolates. This diversity includes variations in virulence, biofilm-forming capacity, and antimicrobial resistance, all of which can significantly influence treatment outcomes. In addition, to minimize hormonal variability and enhance experimental consistency, only male rats were used in this study. However, sex-based differences in immune responses and inflammation are well documented, and thus, the exclusive use of male animals may limit the generalizability of our findings. To enhance translational relevance, future studies should include both clinical MRSA isolates and animals of both sexes to validate and broaden the applicability of LED therapy in MRSA-induced OM. Moreover, the long-term effects of LED therapy, including the potential development of bacterial resistance or recurrence of biofilm formation, were not examined, representing another limitation. Therefore, future studies, particularly human clinical trials, are necessary to determine the optimal treatment parameters and evaluate the long-term safety and efficacy of LED therapy. Finally, investigating the synergistic potential of combining LED therapy with antibiotics may further improve outcomes in the treatment of biofilm-associated infections.

Beyond these findings, additional mechanistic insights are needed to fully understand how LED therapy exerts its anti-biofilm effects. One possible explanation for the additional anti-biofilm effects observed in this study is that LED therapy indirectly modulates biofilm-related gene expression by inducing the production of ROS, which interacts with key bacterial transcriptional regulators, such as *SigB* and *PerR. SigB*, a global stress response regulator, has been implicated in biofilm formation and antibiotic resistance, while *PerR*, as a peroxide-sensing repressor, modulates bacterial responses to oxidative stress [54,55]. Given that LED treatment induces oxidative stress, ROS-mediated signaling could have contributed to the gene expression changes observed in this study. Further investigations involving EPS composition analysis, polysaccharide quantification, or liquid chromatography–mass spectrometry-based metabolic pathway profiling are warranted to elucidate the precise mechanisms by which LED therapy affects biofilm formation. This dual action—disrupting both biofilm structure and its genetic regulation—supports the potential of LED therapy as a promising adjunctive treatment for MRSA-induced OM and other biofilm-associated infections.

In conclusion, this study revealed that red and NIR LED irradiation markedly reduces biofilm formation, decreases biofilm biomass, and downregulates key biofilm-associated genes in MRSA-induced OM. These findings support the potential of LED therapy as a noninvasive and effective approach for managing biofilm-related infections, particularly those that are persistent or refractory to conventional treatments.

## CRediT authorship contribution statement

**Yoo-Seung Ko:** Writing – review & editing, Writing – original draft, Methodology, Investigation, Formal analysis, Data curation, Conceptualization. **Eun-Ji Gi:** Methodology, Investigation, Formal analysis, Data curation. **Sungsu Lee:** Writing – review & editing. **Hong-Chan Kim:** Writing – review & editing, Funding acquisition. **Hyong-Ho Cho:** Writing – review & editing, Supervision, Funding acquisition, Conceptualization.

## Ethics approval and consent to participate

All animal experiments were approved by Chonnam National University's Committee on the Ethics of Animal Experiments (Approval No. CNUHIACUC-21047) and conducted in accordance with the university's Guide for the Care and Use of Laboratory Animals.

## Declaration of competing interest

The authors declare that they have no known competing financial interests or personal relationships that could have appeared to influence the work reported in this paper.

## Figures and Tables

**figs1:**
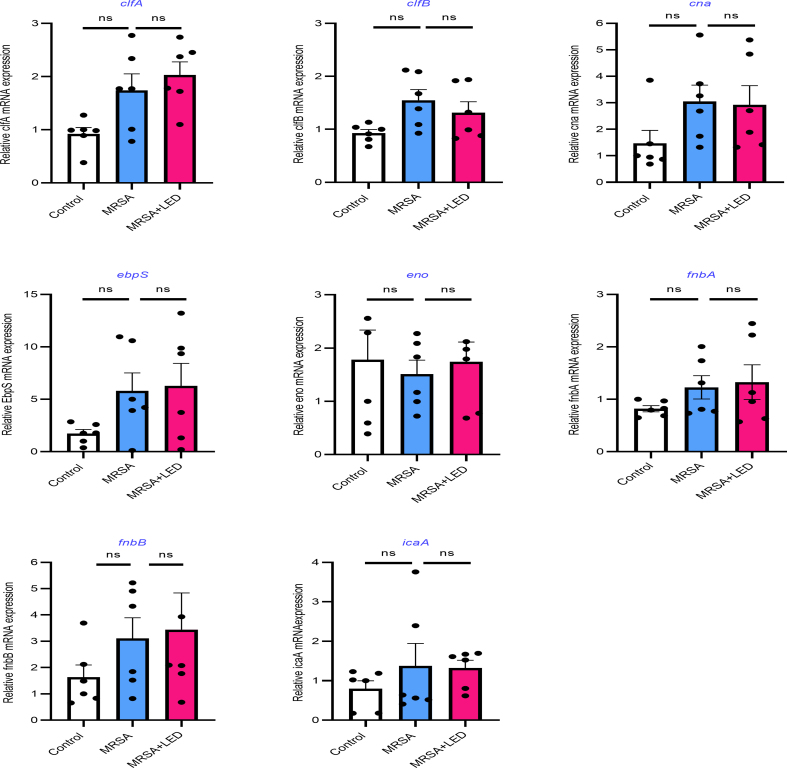


## Data Availability

The raw data supporting the conclusions of this article will be made available by the authors, without reservation.
