# Supplementary material for: Dual red and near-infrared LED therapy inhibits MRSA biofilm in otitis media
Source: Biofilm. 2025 Aug 21;10:100314. doi: 10.1016/j.bioflm.2025.100314 (PMC12398922; doi:10.1016/j.bioflm.2025.100314)
Supplement: Multimedia component 1 [file mmc1.docx]

**Irradiation Parameters**

| Source | Wavelength | Radiant Intensity (W/m^2^) | Exposure Time  (s) | Energy  (J/m^2^) | Fluence  (J/cm^2^) |
| --- | --- | --- | --- | --- | --- |
| Red and  Near-Infrared LED | 655& 842 | 163.2 | 1800 | 293,760 | 29.376 |

**Supplementary Table 1. Irradiation parameters used in experiments, including wavelengths, radiant intensity, exposure time, energy, and fluence.** This table outlines the standardized light settings applied to all animal subjects to ensure consistent and reproducible phototherapeutic effects across experiments.

Suppl Table 1
