# Supplementary material for: Dual red and near-infrared LED therapy inhibits MRSA biofilm in otitis media
Source: Biofilm. 2025 Aug 21;10:100314. doi: 10.1016/j.bioflm.2025.100314 (PMC12398922; doi:10.1016/j.bioflm.2025.100314)
Supplement: Multimedia component 2 [file mmc2.docx]

Target genes and primer pairs used in this study

Target gene Protein function The nucleotide sequence of the primers (5' 3') Product (bp)

*clfA* Clumping- factor A F: ACCCAGGTTCAGATTCTGGCAGCG 165

R: TCGCTGAGTCGGAATCGCTTGCT

*clfB* Clumping- factor B F: AACTCCAGGGCCGCCGGTTG 159

R: CCTGAGTCGCTGTCTGAGCCTGAG

*cna* Collagen binding protein F: AATAGAGGCGCCACGACCGT 156

R: GTGCCTTCCCAAACCTTTTGAGCA

*ebpS* Elastin binding protein F: GGTGCAGCTGGTGCAATGGGTGT 191

R: GCTGCGCCTCCAGCCAAACCT

*eno*  laminin binding protein F: TGCCGTAGGTGACGAAGGTGGTT 195

R: GCACCGTGTTCGCCTTCGAACT

*fib* Fibrinogen binding protein F: CGTCAACAGCAGATGCGAGCG 239

R: TGCATCAGTTTTCGCTGCTGGTTT

*fnbA* Fibronectin-binding protein A F: AAATTGGGAGCAGCATCAGT 121

R: GCAGCTGAATTCCCATTTTC

*fnbB* Fibronectin-binding protein B F: ACGCTCAAGGCGACGGCAAAG 197

R: ACCTTCTGCATGACCTTCTGCACCT

*icaA* Intercellular adhesion A F: GAGGTAAAGCCAACGCACTC 151

R: CCTGTAACCGCACCAAGTTT

*icaB* Intercellular adhesion B F: ATACCGGCGACTGGGTTTAT 140

R: TTGCAAATCGTGGGTATGTGT

*icaC* Intercellular adhesion C F: CTTGGGTATTTGCACGCATT 209

R: GCAATATCATGCCGACACCT

*icaD* Intercellular adhesion D F: ACCCAACGCTAAAATCATCG 211

R: GCGAAAATGCCCATAGTTTC

*16S rRNA* Endogenous control F: GGGACCCGCACAAGCGGTGG 191

R: GGGTTGCGCTCGTTGCGGGA

**Supplementary Table 2. Primer sequences used for qRT-PCR analysis across all experiments**. This table lists all primers applied throughout the study, providing the sequences for each gene analyzed.

Suppl Table 2
